# Age and gender differences in coping and mental health during and post COVID-19 lockdown

**DOI:** 10.1192/j.eurpsy.2022.525

**Published:** 2022-09-01

**Authors:** O. Romano, D. Stout, A. Mendrek

**Affiliations:** Bishop’s University, Psychology, Sherbrooke, Canada

**Keywords:** Gender differences, coping strategies, Anxiety, Depression

## Abstract

**Introduction:**

As a reaction to growing number of COVID-19 cases in Quebec, the government issued a lockdown to prevent further spread of the virus in March 2020. The novelty of the imposed restrictions warranted an assessment of adult coping and potential effects on anxiety and depressive symptoms.

**Objectives:**

The purpose of the present study was to evaluate methods of coping employed during Quebec’s lockdown and their potential ramifications on anxiety and depressive symptoms post-lockdown in Quebec.

**Methods:**

In a retrospective longitudinal design, two-hundred and twenty-three (n = 223) adults (65.5% female; 34.5% male) completed the study online. They were asked to fill out several questionnaires and provide demographic information.

**Results:**

Analysis revealed significant improvement in anxiety symptoms post-lockdown relative to during lockdown across the entire sample. Depressive symptoms also improved significantly across the sample, but the difference was less pronounced among 18–34-year-olds than those 35 and above. Male adults aged 18-34 utilized maladaptive coping strategies to the greatest extent. Moreover, maladaptive coping was significantly associated with anxiety and depressive symptoms and predicted depressive symptoms post-lockdown. Further investigation revealed that young adult males differed from females in their use of substances and self-blame to cope.

**Conclusions:**

Overall, the data suggest that the lockdown adversely affected anxiety and depressive symptoms among the general population. Furthermore, young adults, particularly males, were most susceptible to depressive symptomatology due in part to their methods of coping with the novel context. A follow-up study is warranted. Future studies should also seek to recruit individuals whose self-identified gender is non-traditional (e.g., non-binary).

**Disclosure:**

No significant relationships.

